# Supplementary material for: Loss of histone methyltransferase ASH1L in the developing mouse brain causes autistic-like behaviors
Source: Commun Biol. 2021 Jun 18;4:756. doi: 10.1038/s42003-021-02282-z (PMC8213741; doi:10.1038/s42003-021-02282-z)
Supplement: Supplementary file 2 — Supplementary information [file 42003_2021_2282_MOESM2_ESM.pdf]

.

Supplementary Information

Loss of histone methyltransferase ASH1L in the developing mouse brain causes autistic-like behaviors

Yuen Gao, Natalia Duque-Wilckens, Mohammad B Aljazi, Yan Wu,
Adam J Moeser, George I Mias, Alfred J Robison, Jin He
Correspondence to: hejin1@msu.edu

This PDF file includes:

Supplementary Figure 1 to 6
Supplementary Table 1 to 3

Supplementary Figure 1. Characterization of *Ash1L* knockout mice.

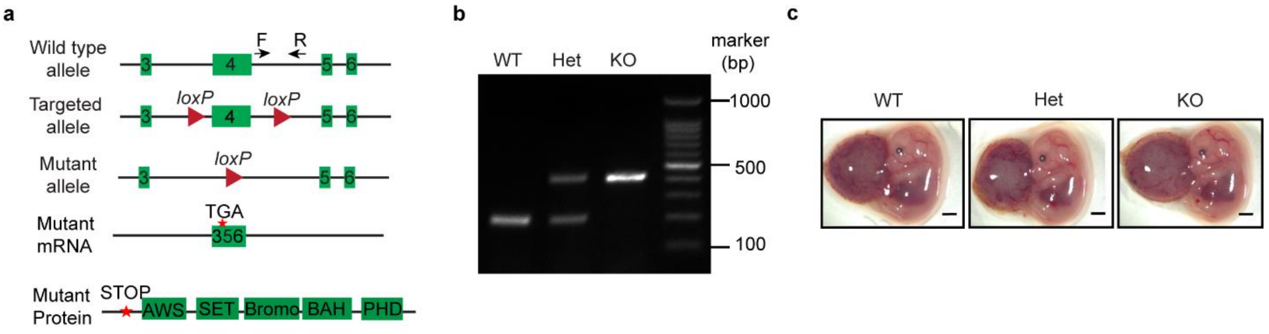

d

Observed (Expected)

	Litters	WT	Het	KO
E16.5	8	11 (12)	25 (24)	12 (12)
P0	26	47 (45.25)	95 (90.5)	39 (45.25)
P21	5	12 (8)	20 (16)	0 (8)

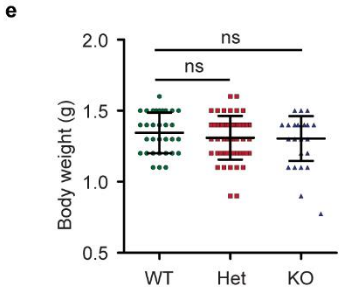

(**a**) Diagram showing the strategy for the generation of *Ash1L* conditional knockout mice. Cre-mediated deletion of exon 4 results in an altered spliced mRNA with a premature stop codon, which generates a truncated protein without all functional AWS, SET, Bromo, BAH and PHD domains. The arrows labeled as F and R represent the genotyping primers. (**b**) Genotyping results showing the PCR results of wild-type, heterozygous, and homozygous *Ash1L* knockout mice. (**c**) Photos showing the gross morphology of wild-type, heterozygous, and homozygous *Ash1L* knockout embryos and placentas at E13.5, bar = 500 µm. (**d**) The genotyping results of global *Ash1L*-KO mice analyzed at embryonic and postnatal stages. (**e**) Body weight of global *Ash1L*-KO mice at P0. For *Ash1L* WT, n = 32 mice; for *Ash1L* Het, n = 53 mice; for *Ash1L* KO, n = 23 mice. *P*-values calculated using a two-tailed *t* test. Error bars in graphs represent mean ± SEM. Note: ns, not significant.

Supplementary Figure 2. Loss of ASH1L delays embryonic and postnatal brain development.

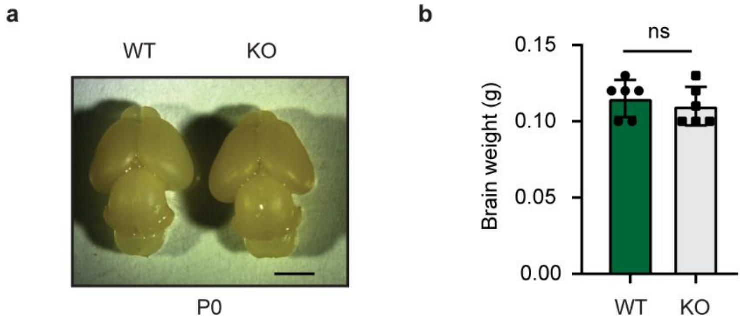

(**a**) Photo showing the gross brain morphology of wild-type and global *Ash1L*-KO mice at P0, bar = 2 mm. (**b**) The brain weight of P0 wild-type and global *Ash1L*-KO mice. n = 6 mice/genotype. *P*-values calculated using a two-tailed *t* test. Error bars in graphs represent mean ± SEM. Note: ns, not significant.

Supplementary Figure 3. Isolation and differentiation of neural progenitor cells *in vitro*.

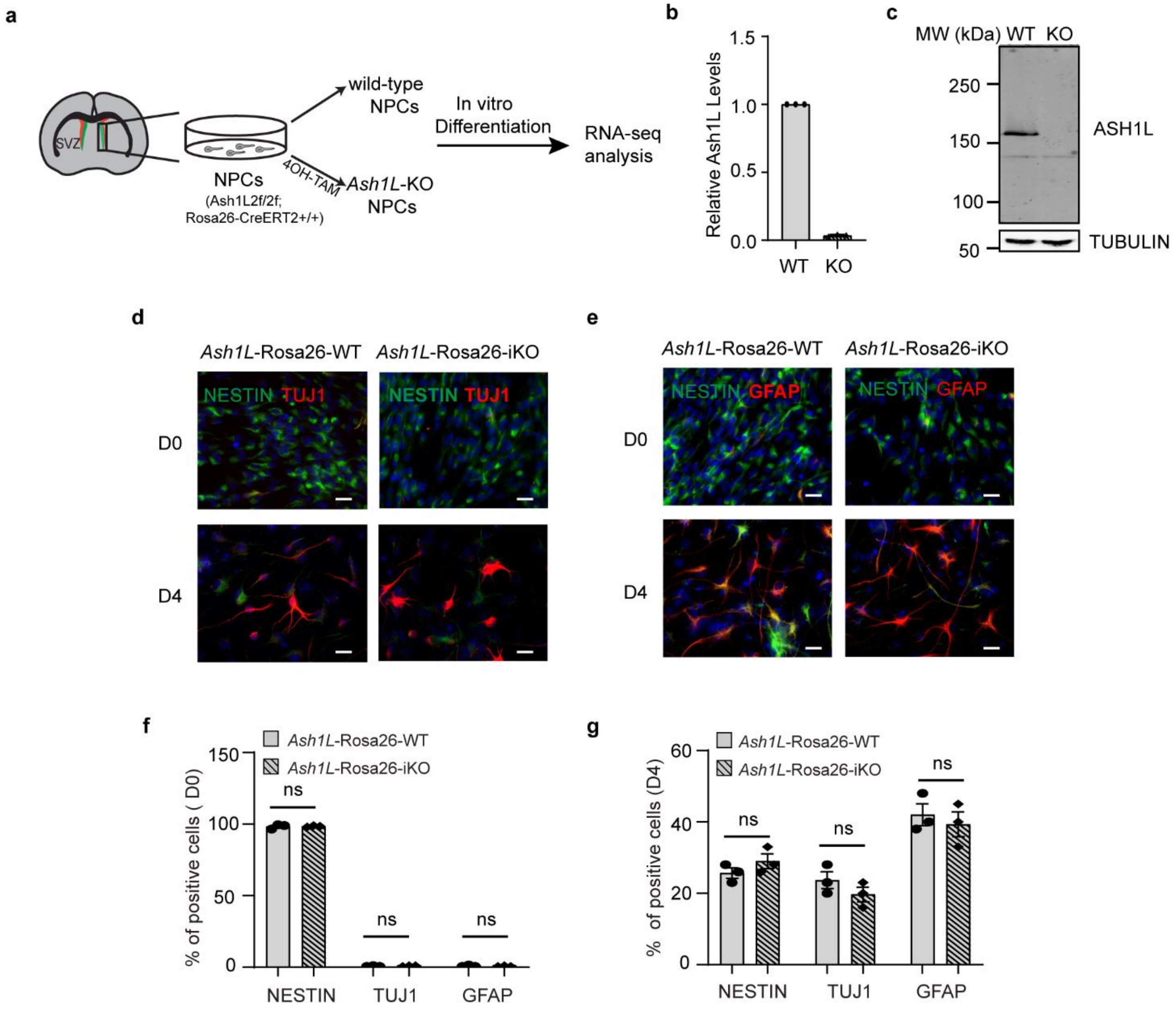

(**a**) Flowchart showing the experimental design for gene expression analysis. (**b**) qRT-PCR analysis showing the expression levels of *Ash1L* in wild-type and knockout NPCs. The results were normalized against levels of *Gapdh* and the expression level in wild-type NPCs was arbitrarily set to 1. The error bars represent mean ± SEM, n = 3 biologically independent samples/genotype. (**c**) Western blot analysis showing the protein levels of ASH1L in wild-type and knockout NPCs. (**d-e**) Photos showing the $NESTIN^{+}/TUJ1^{+}$ (d) and $NESTIN^{+}/GFAP^{+}$ cells (e) at D0 and D4 after induced differentiation, bar = 20 µm. (**f-g**) Quantification of $NESTIN^{+}$, $TUJ1^{+}$, and $GFAP^{+}$ cells at D0 (f) and D4 (g) after induced differentiation. For each group, n = 3 biologically independent samples/genotype. *P*-values calculated using a two-tailed *t* test. Error bars in graphs represent mean ± SEM. Note: $*p < 0.05$; $**p < 0.01$; ns, not significant.

Supplementary Figure 4. Loss of ASH1L impairs expression of genes critical for brain development.

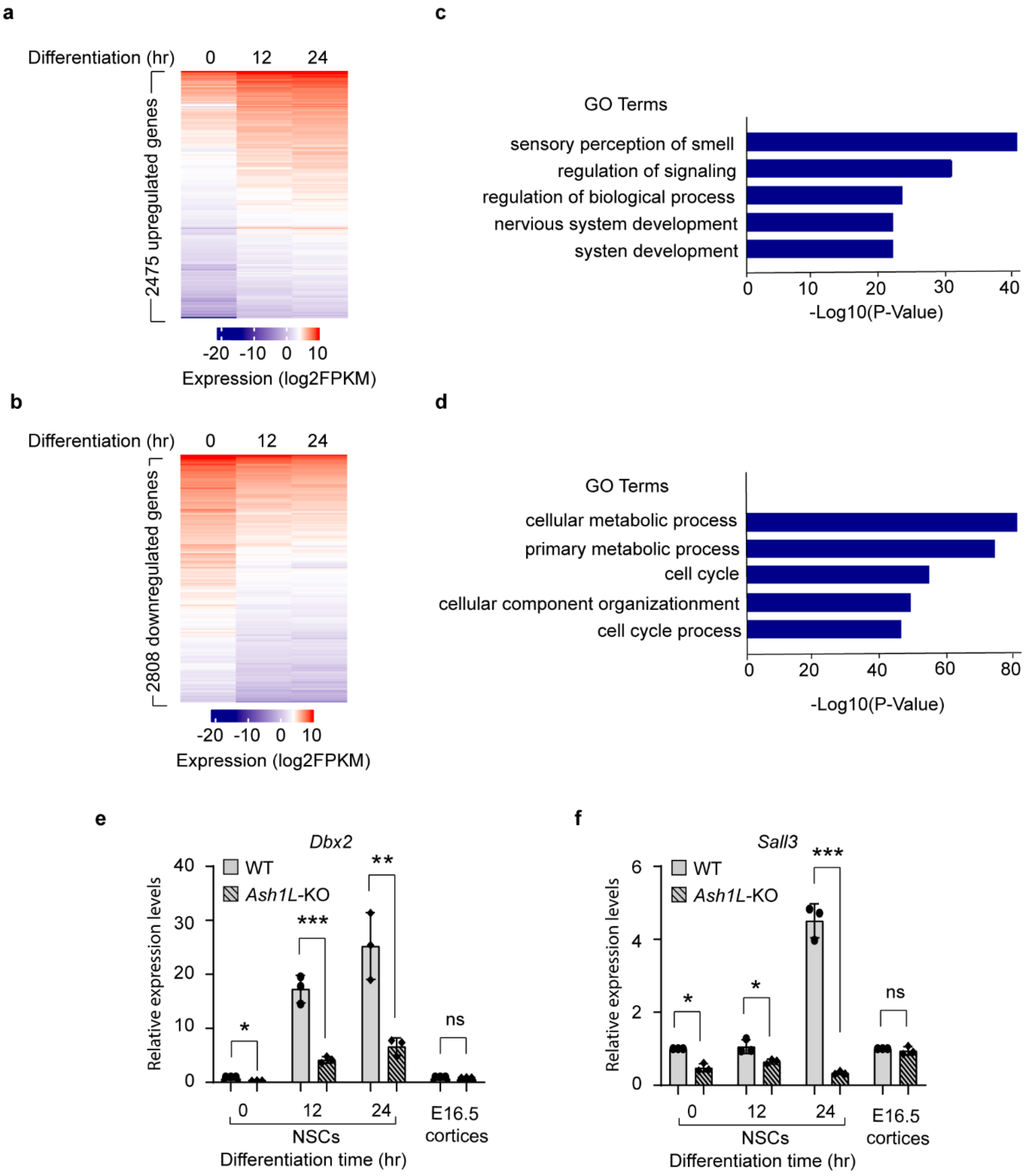

(**a**) Heatmap showing 2,475 upregulated genes in wild-type NPCs during induced differentiation (cutoff: change folds > 1.5, $p < 0.01$). (**b**) Heatmap showing 2,808 downregulated genes in wild-type NPCs during induced differentiation (cutoff: change folds > 1.5, $p < 0.01$). (**c**) Gene ontology enrichment analysis showing the enriched GO terms of 2,475 upregulated genes in the wild-type NPCs during induced differentiation (FDR < 0.05). (**d**) Gene ontology enrichment analysis showing the enriched GO terms of 2,808 downregulated genes in the wild-type NPCs during induced differentiation (FDR < 0.05). (**e-f**) qRT-PCR analysis showing the mRNA levels of *Dbx2* and *Sall3* in wild-type and *Ash1L*-KO NPCs at different time points of induced differentiation and in E16.5 cortices. The results of analysis in NPCs were normalized against levels of *Gapdh* and the

expression level of wild-type NPCs at differentiation time 0 was arbitrarily set to 1. The results of analysis in E16.5 cortices were normalized against levels of *Gapdh* and the expression level of wild-type cortices was arbitrarily set to 1. The error bars represent mean ± SEM, n = 3 biologically independent samples/genotype. *P*-values calculated using a two-tailed *t* test. Note: **P* < 0.05; ***P* < 0.01; ****P* < 0.001; ns, not significant.

Supplementary Figure 5. **Uncropped gel picture for Fig. S1b.**

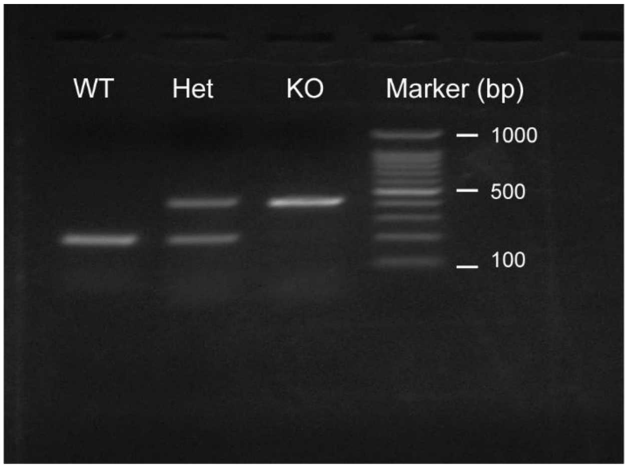

Supplementary Figure 6. **Uncropped gel pictures for Fig. S1c.**

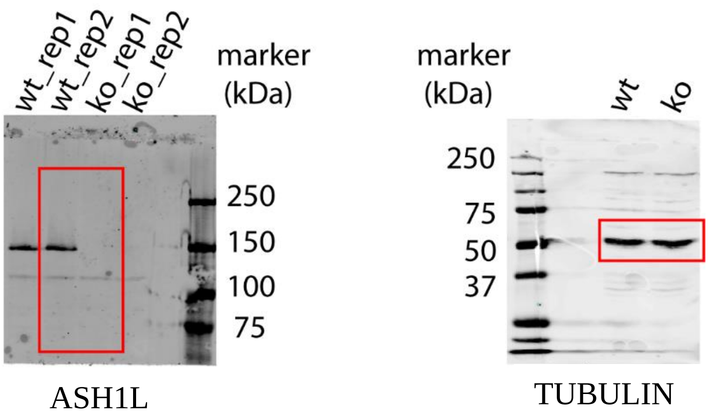

Supplementary Table 1. Result of gene ontology enrichment analysis of 44 genes upregulated in the *Ash1L*-KO NPCs during induced differentiation.

GO biological process complete	P-value	FDR
regulation of developmental growth (GO:0048640)	1.12E-04	4.53E-02
regulation of cell migration (GO:0030336)	1.71E-05	3.36E-02
regulation of locomotion (GO:0040013)	3.20E-06	5.04E-02
regulation of cell motility (GO:2000146)	2.19E-05	3.44E-02
regulation of cellular component movement (GO:0051271)	2.58E-05	3.12E-02
regulation of growth (GO:0045926)	1.49E-04	4.97E-02
regulation of cell growth (GO:0001558)	1.63E-05	3.67E-02
chemotaxis (GO:0006935)	3.69E-05	3.63E-02
plasma membrane bounded cell projection morphogenesis (GO:0120039)	3.93E-05	3.10E-02
taxis (GO:0042330)	3.93E-05	2.95E-02
cell projection morphogenesis (GO:0048858)	4.24E-05	3.04E-02
morphogenesis of an epithelium (GO:0002009)	5.56E-05	3.24E-02
cell part morphogenesis (GO:0032990)	5.76E-05	3.13E-02
tissue morphogenesis (GO:0048729)	2.21E-05	2.90E-02

Supplementary Table 2.Result of gene ontology enrichment analysis of 70 genes down-regulated in the *Ash1L*-KO NPCs during induced differentiation.

GO biological process complete	P-value	FDR
telencephalon development (GO:0021537)	1.18E-06	1.86E-02
cellular process (GO:0009987)	4.38E-06	3.45E-02
exploration behavior (GO:0035640)	4.83E-06	2.54E-02
brain development (GO:0007420)	5.46E-06	2.15E-02
regulation of cell communication (GO:0010646)	7.46E-06	2.35E-02
regulation of signaling (GO:0023051)	7.97E-06	2.10E-02
head development (GO:0060322)	1.17E-05	2.64E-02
locomotory behavior (GO:0007626)	1.30E-05	2.56E-02
animal organ development (GO:0048513)	1.66E-05	2.92E-02
central nervous system development (GO:0007417)	1.68E-05	2.66E-02
synapse maturation (GO:0060074)	2.76E-05	3.95E-02
forebrain development (GO:0030900)	2.95E-05	3.88E-02
regulation of glucose transmembrane transport (GO:0010828)	3.08E-05	3.74E-02
purine ribonucleotide biosynthetic process (GO:0009152)	3.92E-05	4.42E-02

Supplementary Table 3. Primers used in this study.

Name	Sequence (5'-3')	Purpose
GAPDH-F	GCAGTGGCAAAGTGGAGATT	qRT-PCR
GAPDH-R	GAATTTGCCGTGAGTGGAGT	qRT-PCR
Ash1L-F	CCCAGTCAGCTTCCGATAAA	qRT-PCR
Ash1L-R	CTGGAAGGAACTCCATTCACT	qRT-PCR
Dbx2-F	AGGAGTCACAGGTGAAGATTTG	qRT-PCR
Dbx2-R	AACTGTGCTGAAGCTGATCTC	qRT-PCR
Pcdh10-F	AGGTGTATGTCCAAGCCAAG	qRT-PCR
Pcdh10-R	ACTGTGCTGAAGCTGATCTC	qRT-PCR
Emx2-F	CAGAAAGCCAAAGCGGATTC	qRT-PCR
Emx2-R	CCCACCACGTAATGGTTCTT	qRT-PCR
Sall3-F	TCCACTTTCAGAGGCACAAG	qRT-PCR
Sall3-R	GGGCACATTGTCGAGGTATT	qRT-PCR
Foxg1-F	AAGAACGGCAAGTACGAGAAG	qRT-PCR
Foxg1-R	ATAGATGCCATTGAGCGTCAG	qRT-PCR
Nes-cre-F	GCGGTCTGGCAGTAAAAACTATC	Genotyping
Nes-cre-R	GTGAAACAGCATTGCTGTCACTT	Genotyping
Ash1L-WT (floxed)-F	ACTAGCTTTGCTCTCATAAATGTC	Genotyping
Ash1L-WT (floxed)-R	GGGTACATGGAGTTATTAGATCCT	Genotyping
Ash1L-mut-F	CCCACACAAATGTAAGTTTGGA	Genotyping
Ash1L-mut-R	ACATGGAGTTATTAGATCCTG	Genotyping
