# Supplementary material for: Loss of histone methyltransferase ASH1L in the developing mouse brain causes autistic-like behaviors
Source: Commun Biol. 2021 Jun 18;4:756. doi: 10.1038/s42003-021-02282-z (PMC8213741; doi:10.1038/s42003-021-02282-z)
Supplement: Supplementary file 3 — Description of Additional Supplementary Files [file 42003_2021_2282_MOESM3_ESM.pdf]

.

Description of Additional Supplementary Files

File name: Supplementary Data 1

Description: Result of gene ontology enrichment analysis on the upregulated genes in the wild-type NPCs during induced differentiation.xlsx

File name: Supplementary Data 2

Description: Result of gene ontology enrichment analysis on the downregulated genes in the wild-type NPCs during induced differentiation.xlsx

File name: Supplementary Data 3

Description: Source data for graphs.xlsx

File name: Supplementary Movie 1

Description: Posture of wild-type mice upon tail suspension

File name: Supplementary Movie 2

Description: Mild paw clasping of *Ash1L*-Nes-cKO mice upon tail suspension

File name: Supplementary Movie 3

Description: Severe paw clasping of *Ash1L*-Nes-cKO mice upon tail suspension

File name: Supplementary Movie 4

Description: Hair grooming of wild-type mice

File name: Supplementary Movie 5

Description: Hair grooming of *Ash1L*-Nes-cKO mice
